# Ultra-Processed Diets and Pediatric Acne: Insulin–mTOR Activation and Its Potential Role in Cutaneous Inflammation

**DOI:** 10.3390/ijms27167123

**Published:** 2026-08-08

**Authors:** Yasmeen Magharehabed, Zainab Ibrahim, Hesham Shiekhtholth, Nabiha Yusuf

**Affiliations:** 1 School of Medicine, Louisiana State University Health Sciences Center, New Orleans, LA 70112, USA; 2 Heersink School of Medicine, University of Alabama at Birmingham, 1670 University Blvd, Birmingham, AL 35294, USA; 3 Department of Dermatology, University of Alabama at Birmingham, Birmingham, AL 35294, USA

**Keywords:** acne vulgaris, ultra-processed foods, mTORC1, IGF-1, pediatric dermatology, skin microbiome

## Abstract

Acne vulgaris is an inflammatory cutaneous condition that disproportionately affects the pediatric population. While there are several risk factors, emerging evidence suggests that ultra-processed foods (UPFs), as part of Western dietary patterns, may contribute to acne pathophysiology through activation of inflammatory pathways, such as mTORC1. This comprehensive review synthesizes current evidence regarding acne pathogenesis in conjunction with diet-associated inflammatory cascades to better illustrate the potential role of diet as an adjunct to clinical management in pediatric patients. The current literature suggests that increased insulin/IGF-1 levels may contribute to alterations in the skin and gut microbiota. Additionally, components commonly found in UPFs have been proposed to activate the mechanistic target of rapamycin complex (mTORC1) through insulin/IGF-1-dependent and -independent mechanisms, perpetuating inflammatory pathways that give rise to acne pathogenesis. However, the majority of available evidence is derived from adult cohorts; pediatric-specific evidence remains limited. While studies have demonstrated clinical significance in acne severity with dietary interventions, future high-quality studies are required. Through understanding the relationship between diet and acne pathogenesis, future therapeutic and preventative strategies may be developed to improve pediatric acne clinical outcomes.

## 1. Introduction

Acne vulgaris is the eighth most prevalent disease globally, and the second most disabling skin disease, second to eczema [1]. Acne vulgaris affects 231 million people worldwide and accounts for an estimated 117.4 million new cases reported annually [2]. This disease most commonly affects adolescents and young adults, affecting 85% of individuals aged 12–24 years [1]. Prevalence also varies by sex, with female patients being affected earlier in life and male patients often experiencing more severe disease [3]. Several types of acne exist, and they can be characterized by lesion and morphology. Comedonal acne, which consists of open and closed comedones filled with keratin, bacteria, and sebum, is the most common form of acne affecting the pediatric population, with 61% of cases affecting 10–17-year-old children [3]. Acne vulgaris remains clinically significant, particularly for the pediatric population, due to its high prevalence and its incidence during adolescence, a critical period for the formation of self-image, social relationships, and psychosocial development [4]. Acne vulgaris is a multifactorial disease in which hormones, genes, immune dysregulation, and environment all contribute to disease progression. Although diet is not likely the primary cause of acne, diet can exacerbate disease severity and influence inflammatory pathways in individuals that are genetically susceptible [5]. Specifically, diets rich in refined carbohydrates and sugars lead to increased insulin and IGF-1 levels, which hyperactivate lipogenesis and sebogenesis through mTORC1 activation. This has become increasingly relevant with the predominance of ultra-processed foods (UPFs), as defined by the NOVA food classification system, in the Western diet. UPFs are industrial formulations with little intact whole food and are rich in saturated fats and sugars, providing the foundation for hyperinsulinemia and IGF-1 mediated acne development [6,7]. This review aims to summarize the existing scientific knowledge concerning the influence of UPFs on the pathogenesis of acne vulgaris in pediatric and adolescent populations. Prior literature has established the relationship between diet, insulin/IGF-1 signaling, mTOR activation, and acne pathogenesis, particularly through the effects of high-glycemic diets and dairy products [8]. Recent research has expanded this knowledge by UPFs distinctly as a dietary framework with biological influence beyond glycemic load, some of which include gut microbiome interactions and insulin dependent mTOR1 activation [6,9,10]. Additionally, recent studies have shown strain-specific changes in *Cutibacterium acnes*, as well as metagenomic alterations in acne-related skin microbiome [6,11,12]. This review uniquely integrates the UPFs framework with pediatric hormonal changes, adolescent eating behavior, and the gut–skin axis to provide a more comprehensive and informed perspective. Through understanding the implications of UPFs on acne pathophysiology, clinical management can focus on dietary modification as a complementary approach to standard acne treatment, with an emphasis on balanced, sustainable eating patterns.

## 2. Pathogenesis and Immune Responses in Acne

### 2.1. Role of C. acnes and Skin Microbiome Disruption

Various inflammatory pathways can lead to acne, with *Cutibacterium acnes* (*C. acnes*) being the common pathogenic feature of each mechanism. *C. acnes* is a commensal bacterium of the skin microbiome, predominantly colonizing pilosebaceous glands, due to the lipid-rich environment that helps it thrive [13,14]. Its role is often protective, as it produces antimicrobials and neutralizes free radicals through RoxP, protecting the skin barrier from free radical damage [15,16]. *C. acnes* also metabolizes sebum into short-chain fatty acids (SCFAs), which lowers the skin’s pH so that opportunistic bacteria cannot prosper [17]. Unlike other commensal organisms that become pathogenic through increased colonization, *C. acnes* contributes to acne pathogenesis through a loss of phylotype diversity and the predominance of pro-inflammatory strains. *C. acnes* has six main phylotypes, with IA1 and IA2 being the main phylotypes found on healthy human skin [11]. An in vitro study examining *C. acnes* phylotypes in healthy skin and acne lesions found that the IA1 phylotype contributed to 42% of the microbiome population in healthy skin. In contrast, IA1 accounted for 86–92% of *C. acnes* phylotypes of acne lesions, emphasizing that acne is associated with a loss of phylotype diversity, rather than increased bacterial load [11,18].

Furthermore, IA1 strains also propagate immunogenic activity through biofilm formation, establishing bacterial persistence and enhanced virulence [18]. These biofilms confer resistance to antibiotics and benzoyl peroxide, key treatments for severe comedonal acne [19,20]. This microbiome dysbiosis is also perpetuated through structural and physiologic changes, such as follicular plugging. Follicular plugging creates a low-oxygen environment in which *C. acnes* predominates, producing SCFAs that inhibit histone deacetylases (HDACs), enzymes that ordinarily help regulate the skin’s immune response. HDAC inhibition promotes dysregulated cutaneous inflammatory signaling, promoting erythema and swelling at the skin barrier. Furthermore, *C. acnes* colonization is closely tied to pubertal changes, particularly androgen excess, which drives sebum overproduction. This lipophilic follicular environment supports *C. acnes* colonization, creating a cycle of microbiome disruption, inflammation, and acne development. Diet composition also alters the skin microbiome, which will be explored in detail throughout this review.

### 2.2. Innate and Adaptive Immune Activation

Microbiome dysbiosis supports innate and adaptive immune activation. The innate immune system contributes to acne pathogenesis through the recognition of *C. acnes* by toll-like receptor 2 (TLR2) on skin-resident cells, including keratinocytes, macrophages, and sebocytes [21]. TLR2-expressing macrophages surrounding pilosebaceous follicles are subsequently activated [22]. In addition, *C. acnes* derived free fatty acids activate both TLR2 and toll-like receptor 4 (TLR4) [22]. Activation of these receptors, together with the recognition of *C. acnes* peptidoglycan by innate immune cells, activates three crucial downstream pathways. The NF-κB, AP-1, and MAPK pathways are induced, leading to pro-inflammatory cytokine release, disruption of the dermal matrix, and amplified immune responses [19,20,21,22]. The NF-κB pathway promotes transcription of IL-1β, IL-6, IL-8, and TNF-α in skin-resident cells, promoting follicular hyperkeratinization, neutrophil recruitment, and cytokine amplification. Through AP-1, metalloproteinase (MMP) release coordinates tissue remodeling. Importantly, TLR2 activation also induces calcium entry into keratinocytes, leading to calcium-dependent keratinocyte differentiation and keratin 1 expression. TLR2-mediated calcium influx further contributes to follicular hyperkeratinization and amplification of local inflammation [23].

Aside from the TLR pathway, *C. acnes* also triggers the activation of NLRP3 inflammasome, a multiprotein complex that cleaves pro-IL-1β and pro-IL-18 into IL-1β and IL-18, respectively. These inflammatory cytokines lead to production of other cytokines, neutrophil recruitment, and follicular hyperproliferation, perpetuating *C. acnes* overgrowth and the continuation of this cycle [19,24]. NLRP3 activation also leads to the cleavage of gasdermin D (GSDMD), allowing for pore formation within the cell membrane, triggering pyroptosis. This form of cell death leads to cytokine release within the extracellular space [25,26]. Alongside these innate mechanisms, the adaptive immune system contributes to acne pathogenesis through the production of Th1 and Th17 lymphocytes. Dendritic cells recognize *C. acnes* phylotype IA1 and produce IL-1β, IL-6, and IL-23, which promote T-cell polarization into Th1 and Th17. These differentiated T cells produce the cytokines IL-17A and IFN-γ, activating inflammatory cells like macrophages and neutrophils [18]. These innate and adaptive immune responses are further potentiated by dietary and metabolic signals which converge through the mTORC1 pathway, establishing a self-amplifying cycle of hormonal dysregulation and immune activation.

## 3. Metabolic and Molecular Pathways in Acne

### 3.1. Dietary Influences on Acne Pathogenesis

Diet contributes to acne pathogenesis through various mechanisms. Omega-6 and Omega-3 fatty acid imbalances are considered to be strongly involved in acne development [27]. Omega-3 fatty acids are known for their anti-inflammatory activity through inhibiting TLR1/TLR2 dimerization and NLRP3 inflammasome activation. Dairy products, refined carbohydrates, chocolate, and saturated fats can exacerbate acne by activating alimentary metabolic cues. Saturated fatty acids induce inflammation through TLR2/IL-1B receptor expression promoting Th17 lymphocyte differentiation. This enhances IL-17A secretion, which promotes keratinocyte hyperproliferation [28]. Furthermore, increased consumption of dairy products and high glycemic index are associated with increased insulin levels and elevated IGF-1 levels, resulting in excessive sebum production.

### 3.2. Insulin, IGF-1, and IGFBP-3 Signaling

High glycemic diets can promote massive spikes in blood glucose, elevating insulin, which subsequently increases IGF-1 levels. In addition to diet, IGF-1 activity is also influenced by physiologic factors including age, sex, pubertal stage, growth hormone secretion, nutritional status, hepatic function, and body composition. Under normal conditions, insulin-like growth factor binding protein-3 (IGFBP-3) sequesters the majority of circulating IGF-1, thereby regulating the amount of free, biologically active IGF-1 available to bind to receptors and exert its anabolic effects. With high glycemic load and subsequent hyperinsulinemia, hepatic IGFBP-3 production is reduced, and serine protease activity against IGFBP-3 increases, allowing a significant amount of IGF-1 to remain unbound and bioavailable [29,30]. However, these findings are derived primarily from studies in patients with diabetes and may not be fully generalizable to otherwise healthy pediatric populations. At the cutaneous level, hyperinsulinemia suppresses keratinocyte IGFBP-3 production, further increasing local IGF-1 signaling and promoting keratinocyte proliferation and follicular hyperkeratinization [2]. Decreased IGFBP-3 also is associated with decreased modulation of retinoid X receptor-α (RXRα) mediated signaling in the skin. IGFBP-3 and RXRα bind together to form the IGFBP-3-RXRα complex, which regulates follicular proliferation and promotes regular turnover. With a decrease in the synergistic effect of IGFBP-3-RXRα complex, follicular cells undergo uncontrolled proliferation and promote the comedogenic environment that favors *C. acnes* colonization. Clinically, increased serum IGF-1 levels are present in patients with acne and directly correspond with increased acne severity [31]. Elevated insulin and IGF-1 levels also activate pro-lipogenic transcription factors through phosphoinositide 3-kinase (PI3K) and Akt activation, which will be explored in the next section [32,33].

### 3.3. PI3K/Akt Pathway and SREBP-1 Activation Through FoxO1 Suppression and mTORC1 Hyperactivation

**Figure 1:**
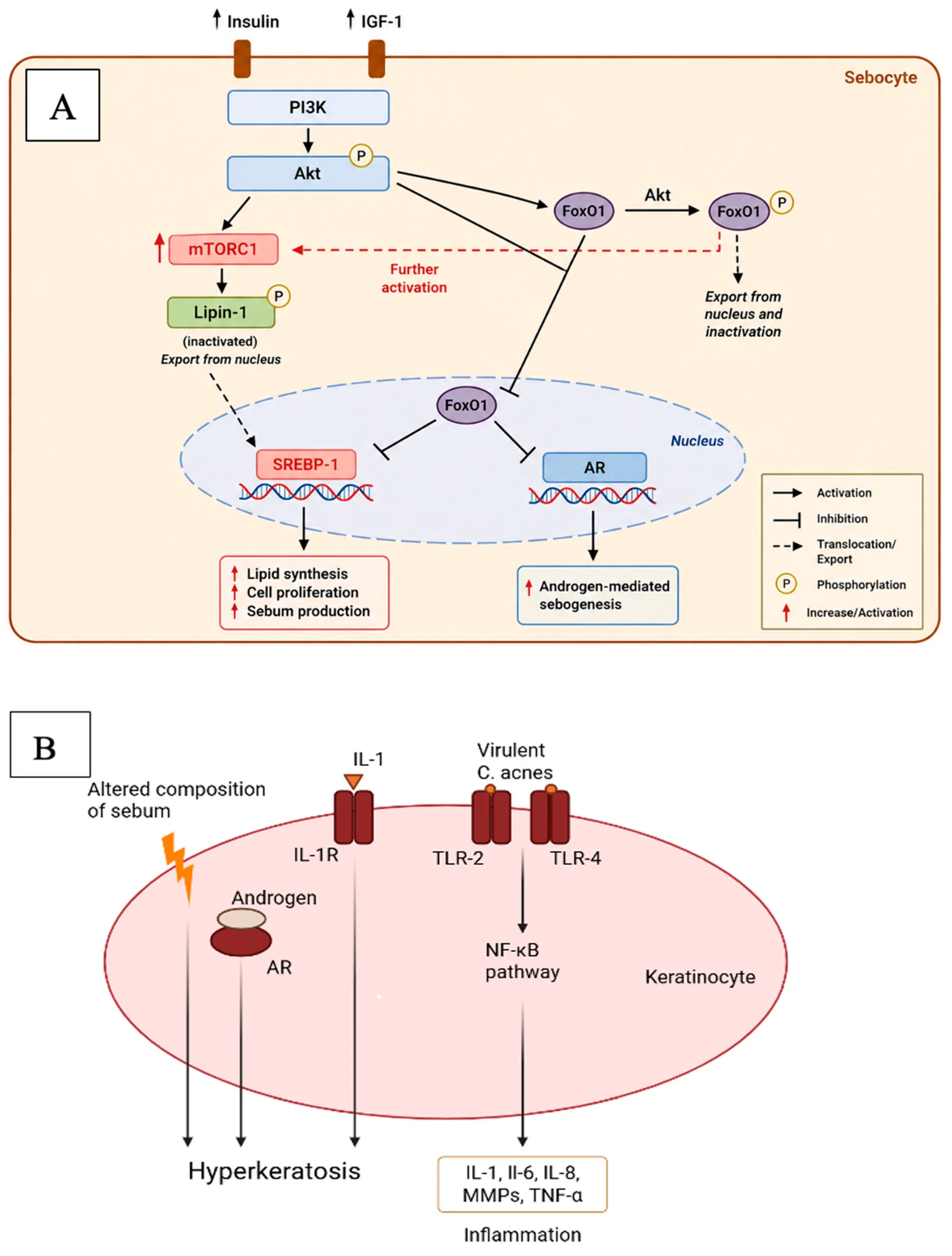
Evidence-supported signaling pathways implicated in acne pathogenesis. (**A**) High insulin/IGF-1 signaling activates PI3K, which subsequently activates Akt through phosphorylation. Akt enhances SREBP-1 activity through two complementary mechanisms. Akt activates mTORC1, resulting in lipin-1 inactivation and increased SREBP-1 transcriptional activity. Akt also phosphorylates FoxO1, promoting its nuclear export and relieving its inhibitory effects on androgen receptor (AR)-mediated sebogenesis, SREBP-1, and mTORC1. Together, hyperactivated mTORC1 and suppressed FoxO1 increase SREBP-1 activity, promoting lipid synthesis, cell proliferation, and sebum production. (**B**) Evidence-supported cellular pathways implicated in acne pathogenesis. Altered sebum composition, androgen signaling, and IL-1 contribute to follicular hyperkeratosis. Virulent *Cutibacterium acnes* is recognized by TLR2 and TLR4, activating the NF-κB pathway and promoting downstream inflammatory cytokine production. These figures were created using Biorender betweeen 10–12 June 2026

With a high glycemic index diet, increased circulating insulin and IGF-1 bind to receptors on sebocytes mediating PI3K activation, which subsequently activates Akt through phosphorylation [34,35]. The major pathogenic mechanisms underlying pediatric acne are summarized in Figure 1A. Once the PI3K/Akt pathway is induced, Akt induces the expression of SREBP-1, a transcription factor that controls fatty acid and lipid production within cells [33]. Akt achieves this altered transcription activity through two mechanisms. Akt directly activates mTORC1, through phosphorylation and inactivation of the TSC1/TSC2 complex, which relieves inhibition of the small GTPase Rheb, a key amplifier of sebum production and direct activator of mTORC1 [36,37]. Hyperactivated mTORC1 phosphorylates lipin-1, promoting its exclusion from the nucleus and subsequent inactivation. The inactivation of lipin-1 allows increased SREBP-1 activation and activity, which was previously controlled by lipin-1 [38]. mTORC1 also activates S6K1, which processes SREBP-1 into its active form, allowing for the translation of lipogenic genes [39]. In addition, mTORC1 upregulates PPARγ and activates HIF-1α, promoting sebocyte differentiation, increasing lipid synthesis, and reducing lipid catabolism. Together, these pathways amplify sebum production and contribute to acne pathogenesis [38,40]. The molecular pathways involved in sebocyte and keratinocyte dysregulation are illustrated in Figure 1B.

Akt also indirectly activates SREBP-1 through FoxO1 repression. Akt phosphorylates FoxO1, a regulatory transcription factor, exporting it from the nucleus and removing its regulatory effects on sebogenesis [34]. FoxO1 repression leads to the activation of several pathways that promote sebum formation and acne pathogenesis. Once FoxO1’s regulatory effects is removed from Androgen Receptor (AR) transcription, this increases sensitivity to androgens and upregulates androgen-related sebogenesis through increased AR transcriptional activity. FoxO1 regulation of SREBP1-driven lipogenesis is also lost, further perpetuating lipogenesis that is already underway through the mTORC1/Akt pathway [38]. Additionally, diminished regulation of PPARγ and LXRα leads to increased lipid uptake and cholesterol metabolism by sebocytes [38]. Importantly, phosphorylation-induced nuclear export of FoxO1 also relieves its inhibitory effects on mTORC1, amplifying the initial mTORC1 hyperactivation induced by Akt.

Through indirect and direct mechanisms, SREBP-1 activity is enhanced. SREBP-1 translocates from its usual location in the endoplasmic reticulum into the nucleus, activating a series of crucial genes. SREBP-1 nuclear translocation activates lipogenic enzymes, including fatty acid synthase (FASN), stearoyl-CoA desaturase (SCD), Δ6-desaturase (FADS2), and acetyl-CoA carboxylase, collectively leading to increased monounsaturated fatty acid synthesis [33]. Consequently, SREBP-1 activation can also suppress FoxO1 directly, promoting its own continuous activation [41,42].

Significantly, the removal of FoxO1 and consequent amplification of SREBP-1 and mTORC1 facilitates increased sebum, which is characterized by an altered lipid composition [33,38,39]. The resulting sebum composition consists of increased monounsaturated fatty acids (MUFAs), sapienic acid, and oleic acid. With the increase in MUFAs, linoleic acid is reduced, which is a crucial essential polyunsaturated fatty acid that reduces *C. acnes* proliferation by reducing sebum viscosity and inhibiting keratinization [43,44]. Furthermore, mTORC1 activation directly leads to increased levels of saturated free fatty acids, such as palmitic acid, which directly activate the downstream inflammatory cascades, such as NLRP3 inflammasome and TLR2 activation leading to increased acne severity [31,45]. Furthermore, increased bioavailable IGF-1 also amplifies androgen-controlled acne proliferation. Androgens alone do not create a robust response in activating the mTOR pathway. Androgens promote mTOR activation only in conjunction with IGF-1, emphasizing that increased IGF-1 is crucial for hormone-mediated mTOR activation and resulting cutaneous inflammation.

## 4. Ultra-Processed Diets: Definition and Metabolic Impact

### 4.1. Nutritional Composition

Western diets are considerably different from those of non-Western diets. Among different countries in the West, there is congruence between relatively higher fat and sugar contents in food items [46]. Western diets are deficient in omega-3 fatty acids and have excessive amounts of omega-6 fatty acids compared to their non-Western counterparts [47]. Together, these dietary differences contribute to the greater prevalence of acne in Western populations. Western diets now include ultra-processed foods (UPFs), which are formulations derived from foods and additives not usually used in culinary preparations that contain little intact food. The concept of UPFs was created in the NOVA classification, which divided food into four groups based on the extent and purpose of food processing: unprocessed (unPF) and minimally processed foods (MPF), processed culinary ingredients, processed foods (PF), and ultra-processed foods (UPF) [6,48]. The majority of UPFs are marketed to younger generations and include sugary drinks, packaged snacks, fast food, and ready-to-eat meals [49]. The biological effects by UPFs are likely to reflect a combined influence of many nutritional components, rather than a single dietary exposure. UPFs are prevalent in pediatric diets, with National Health and Nutrition Examination Survey (NHANES) data from 2009–2014 revealing that 67% of total energy intake in US children’s diet comes from UPF intake [49]. This food group often has a suboptimal nutritional profile, being energy dense and containing a high content of saturated fats, salts, and sugars [6]. UPFs are also typically high in refined carbohydrates and include protein sources such as hydrolyzed proteins, soya protein isolate, gluten, casein, and whey protein [50,51]. Colors, flavors, emulsifiers, and other additives are frequently added to make the final product more palatable [51]. The nutrient profile and additives contribute to dysglycemia, activating mTORC acne pathogenesis through several mechanisms (Table 1).

### 4.2. UPF-Induced Dysglycemia and Insulin-Independent mTORC1 Activation

Interestingly, UPFs is associated with dysglycemia, rather than hyperglycemia. Rather than simply elevating blood glucose levels, UPFs promote glucose intolerance and insulin resistance, driving metabolic disruption [52]. With the disruption of the natural structure of foods, UPFs become rapidly absorbed within the gastrointestinal tract, giving rise to rapidly elevated blood glucose levels with secondary increased insulin levels [53]. This enhanced post-prandial hyperinsulinemia and increased IGF-1 also results in decreased IGFBP-3 levels, with increased IGF-1 bioavailability. In recent studies, foods with rapid GI absorption, such as instant mashed potatoes, were associated with decreased chronic IGFBP-3 levels, whereas foods with slower GI absorption indicated increased IGFBP-3 over time [54,55]. Circulating IGF-1 initiates the PI3K/Akt/mTOR cascade, increasing sebogenesis and acne formation.

UPFs also contain increased amounts of whey and dairy protein, which can activate mTORC1 through hormonal signals with insulin and IGF-1, and with nutrient signaling. Increased milk dairy protein consumption is associated with increased endogenous IGF1-level, due to branched chain-amino acids that activate downstream inflammatory pathways independent of glucose levels [56]. These branched-chain amino acids, particularly leucine, can also activate mTORC1 independent of insulin and IGF-1 [56]. Leucine is responsible for milk/dairy protein’s secondary activation of mTORC1 through its influence on Sestrin2, an intracellular leucine sensor [57,58]. With high levels of leucine, Sestrin2 activates a series of GTPase-activating proteins that ultimately lead to the translocation of mTORC1 to the lysosomal surface. Within this lysosome, Rheb-GTP, the final activator of mTORC1, is able to activate mTORC [59,60,61]. Through multiple converging pathways that promote mTOR activation, milk and UPFs give rise to increased mTOR hyperactivation and subsequent inflammatory features of acne.

### 4.3. UPF-Induced Gut Microbiome Dysbiosis and the Gut–Skin Axis

UPFs also contribute to gut dysbiosis, leading to inflammation and cutaneous manifestations. UPFs contain emulsifiers, including polysorbate 80 (P80) and carboxymethylcellulose (CMC), to enhance food shelf life and to give foods a smoother, rich consistency [62]. These emulsifiers directly damage the gut mucosa, through thinning the protective mucus layer and altering tight junctions that usually prevent pathogens from invading the epithelial layer [9]. Together, these effects result in increased entry of invading pathogens across the gut wall and into the bloodstream. Emulsifiers are also able to activate the NF-κB cascade, resulting in TNF-α and cytokine production that promotes further inflammation [9]. Both emulsifiers also lead to increased leakage of flagellin and lipopolysaccharide (LPS), which may both activate an inflammatory response [63]. Impaired intestinal barrier function allows for increased LPS to enter circulation and may contribute to cutaneous inflammation, including within pilosebaceous units [64,65]. This may promote mTORC-1 independent inflammatory pathways that contribute to cutaneous inflammation. Furthermore, the fiber-deficient nature of UPFs leads to gut microbiome consumption of the gut mucus glycoprotein layer for nutrients and energy, perpetuating weakened gut integrity [9].

Western diets also promote alterations in the gut microbiome. Animal studies have suggested that gut microbial metabolites, such as SCFAs, may influence IGF-1 production by hepatic and adipose tissue [10]. However, these findings have not been directly demonstrated in pediatric patients. SCFAs work partly through GPR41 and GPR43 receptors, tying gut microbial activity directly into the body’s growth signaling, and forming a gut-skin feedback loop [10,66]. UPF-induced gut dysbiosis is associated with reduced populations of SCFA-producing bacteria, resulting in altered SCFA production and impaired intestinal barrier function [65]. In parallel, UPFs may promote insulin/IGF-1 signaling through their high glycemic load and insulinogenic properties, contributing to increased sebaceous gland activity and changes in sebum composition that favor *C. acnes* IA1 phylotypes. These inflammatory phylotypes have been associated with enhanced local IGF-1/IGF-1R signaling, which may further amplify sebogenesis through the same cascade described in Section 3.3 [65]. Notably, SCFAs appear to exert context-dependent effects rather than acting uniformly as harmful or protective signals. Although SCFAs exhibit context-dependent biological effects, butyrate supports epidermal barrier integrity by enhancing keratinocyte mitochondrial metabolism and structural protein synthesis, while also modulating cutaneous immunity through HDAC inhibition and regulatory T-cell expansion [67,68]. When UPF-induced dysbiosis reduces overall SCFA-producing bacterial populations, this protective signaling weakens, reducing barrier function and limiting anti-inflammatory immune regulation while the IGF-1-driven sebogenic pathway may be independently affected. This duality underscores that SCFA signaling reflects a complex, tissue-specific balance that warrants further mechanistic clarification. Furthermore, the artificial sweeteners of UPFs, including sucralose, saccharin, and acesulfame-K, exert bacteriostatic effects that favor pathogenic microbiota like Enterobacteriaceae to prosper and further damage the gut lining [62,69].

### 4.4. UPF-Induced Skin Microbiome Alteration

In addition to dysglycemia and gut-microbiome disruption, UPFs may alter the skin microbiome through indirect metabolic and inflammatory mechanisms. As outlined throughout Section 4, UPFs have been proposed to promote increases in insulin and IGF-1, leading to mTOR activation and subsequent activation of FADS2 and SCD1 [31,38]. This alters sebum composition to favor *C. acnes* phylotype IA1 predominance [18,65]. Furthermore, a 2025 metagenomic study indicated that acne-affected skin contained higher levels of S. epidermidis that directly correlated with elevated triglyceride composition [12]. Furthermore, acne-affected skin also contains higher amounts of *C. granulosum* and lower levels of anti-inflammatory microbiota [70].

Increased *C. acnes* phylotype IA1 efficiently forms adhesive biofilms that have been associated with reduced susceptibility to benzoyl peroxide and tetracyclines [18,71]. Notably, UPFs also drive gut dysbiosis and a secondary decrease in SCFA production through reduced fermentable fiber availability. High fiber diets are associated with significantly decreased antibiotic resistance genes, which are mediated through increased SCFA production [72]. Together, these findings suggest that UPF consumption may contribute to microbial changes associated with antibiotic resistance through bacterial biofilms on the skin and gene alterations within the gut.

**Table 1:** Proposed mechanisms by which ultra-processed food (UPF) components contribute to acne pathogenesis.

UPF Component	Mechanism	Downstream Effect	Evidence Type	Reference
Refined carbohydrates (high glycemic load)	Hyperinsulinemia → ↓IGFBP-3 → ↑free IGF-1	PI3K/Akt/mTORC1 activation, increased sebogenesis	Clinical	[31,73,74]
Dairy/whey protein	Direct hepatic IGF-1 stimulation	PI3K/Akt/mTORC1 activation	Epidemiologic	[1,31,75]
Leucine (whey-derived)	Sestrin2/GATOR2/Rag GTPase pathway	Direct mTORC1 activation (TSC-independent)	Cell-based	[76,77,78]
Saturated fatty acids	TLR2/TLR4 activation, Th17 polarization	NF-κB activation, IL-17A production, keratinocyte hyperproliferation	Cell-based	[23,38,79]
Emulsifiers (P80, CMC)	Gut barrier disruption, ↑LPS translocation	Systemic inflammation, gut–skin axis dysregulation	Cell-based	[9,80,81]
Artificial sweeteners	Gut microbiome dysbiosis	Altered SCFA production, inflammation	Clinical	[69,82]
Omega-6 excess/Omega-3 deficiency	↑Pro-inflammatory eicosanoids, ↓TLR1/TLR2 inhibition	NLRP3 activation, sustained inflammation	Genetic (Mendelian randomization)	[83,84,85]

↑ increase; ↓ decrease; → leads to.

## 5. Evidence Linking Diet and Acne

### 5.1. Epidemiologic Observations and Interventional Studies

Non-Western populations have demonstrated significantly decreased acne rates in their populations. For example, a study done by Cordain et al. compared civilians of Papua New Guinea and Paraguay, aged 15 to 25 years, with zero cases of acne found in either population [86]. In a comparable Western population, approximately 120 cases of facial acne were predicted. Both examined groups consumed unprocessed, low-glycemic diets with minimal dairy. While genetics can serve as a confounding variable, the study noted similar acne rates in populations with similar ethnic backgrounds, but more Westernized civilizations [86].

Experimental models are crucial in understanding the real-world impact of diet alterations on acne progression (Table 2). Due to limited direct pediatric clinical studies evaluating UPFs, Table 2 includes representative adult clinical studies, providing context for current clinical evidence. In one randomized clinical trial, Smith et al. implemented low-glycemic load diets for 12 weeks in individuals aged 15–25 years. This study demonstrated a significant reduction in acne lesions and improved insulin sensitivity (*p* = 0.03) [87]. In another study focused on adult patients, low glycemic index diets resulted in significantly decreased IGF-1 levels (*p* = 0.049). Although these findings suggest a potential mechanistic role for modification of diet, caution should be taken when inferring results from adult populations to pediatric populations, as age-related biological differences may influence response to treatment, as well as disease pathophysiology [88]. Furthermore, meta-analyses have emphasized the significance of the gut microbiome on inflammatory acne. Oral probiotics significantly improved inflammatory and non-inflammatory acne lesions, with varying acne reduction with different probiotic strains [89,90]. Overall, the available clinical evidence remains limited, particularly in pediatric populations. Differences in dietary assessment, study populations, and intervention design limit the direct comparison across different studies. Furthermore, most intervention studies have evaluated individual dietary patterns other than UPFs as a specific exposure. Consequently, direct clinical evidence on UPFs on pediatric populations remains limited, despite the available evidence supporting plausible links between diet and acne.

### 5.2. Western vs. Non-Western Skin Microbiomes

UPFs alter the skin microbiome, through various pathways. Studies have demonstrated this effect by examining Western and non-Western societies’ skin microbiomes. In one study, an indigenous Yanomami community in the Amazon was found to have a complex skin microbiome, with 115 unidentified microbiotas [91]. Through analyses, these microbiotas were found to aid in skin barrier protection, through lipid metabolism and promotion of an acidic environment, in which pathogens struggle to grow [91]. Notably, the study also examined Western travelers, who transiently acquired the Yanomami skin microbiome during their stay in the Amazon and returned to their baseline microbiome upon returning to industrialized cities [91]. In another study examining Korean diet patterns, dietary patterns associated with higher contents of whole foods and vegetables showed lower levels of *C. acnes* and greater microbiome complexity, compared to individuals with high fast-food consumption [92]. These differences suggest that the skin microbiome composition is highly variable and influenced by environmental and lifestyle factors.

### 5.3. Limitations of Current Evidence

While evidence supporting a diet-dependent role in acne pathogenesis is growing, several limitations should be acknowledged, one being recall bias. Many of these studies require participants to recall their dietary habits, and, because many are observational in nature, they are inherently subject to recall bias [93,94]. Another limitation is the possible confounding variable of weight loss. Alterations in diet often result in caloric restriction and an overall reduction in glycemic load, which may improve insulin sensitivity and subsequently reduce acne severity independent of specific dietary components [87]. There is also the confounding variable of age and sex. After the teenage years, women are more often affected by acne than men. The overall prevalence of acne declines with age in both sexes [95]. UPF intake may also reflect an overall lifestyle pattern rather than an independent causal factor. Busy schedules and quick meal management can drive UPF consumption [96]. Additionally, there is a relatively short duration of these clinical trials. Many studies have only lasted 12 weeks, in which longer clinical intervention may be required to see the full effects of dietary intervention [88]. In addition, the 2024 AAD guidelines revealed that the available evidence is conflicting. One randomized control trial of 84 patients evaluating a low glycemic diet in combination with benzoyl peroxide found no significant differences in acne lesion counts [97]. Despite these limitations, the current evidence proposes a possible role that diet may have on acne pathogenesis, although further high-quality studies are required to guide further management.

**Table 2:** Key interventional studies evaluating dietary and microbiome-targeted therapies for acne vulgaris.

Study	Population	Intervention	Duration	Key Outcome	*p*-Value	References
Smith et al. (2007) *Low-glycemic-load diet RCT*	43 male acne patients (15–25 years)	Low-glycemic-load diet vs. control diet	12 weeks	↓Acne lesions; ↑insulin sensitivity	*p* = 0.03	[87]
Burris et al. (2018) *Low glycemic-load dietary intervention*	66 Acne patients	Low-glycemic-load dietary intervention	2 weeks	↓IGF-1 concentrations. No clinical acne outcomes measured	*p* = 0.049	[88]
Lin et al. (2025) *Systematic review and meta-analysis of oral probiotics for acne*	623 patients (9 RCTs)	Oral probiotics vs. placebo/standard therapy	4–12 weeks	No effect at 4 weeks; improvement in acne severity and lesion counts at 12 weeks.	Significant	[89,90]
Reynolds et al. (2024) *AAD guideline summary of multiple low-glycemic-load diet RCT*	RCTs of 45, 32, 43, and 84 acne patients	Low-glycemic-load diet vs. high-GL or usual diet	8–12 weeks	3 of 4 RCTs showed significant benefit 1 RCT (n = 84, low-GL + BP 2.5% gel) showed no significant difference	NS	[97]

↑ increase; ↓ decrease; → leads to.

## 6. Pediatric-Specific Considerations

### 6.1. Hormonal Impacts: Puberty and the IGF-1 Surge

**Figure 2:**
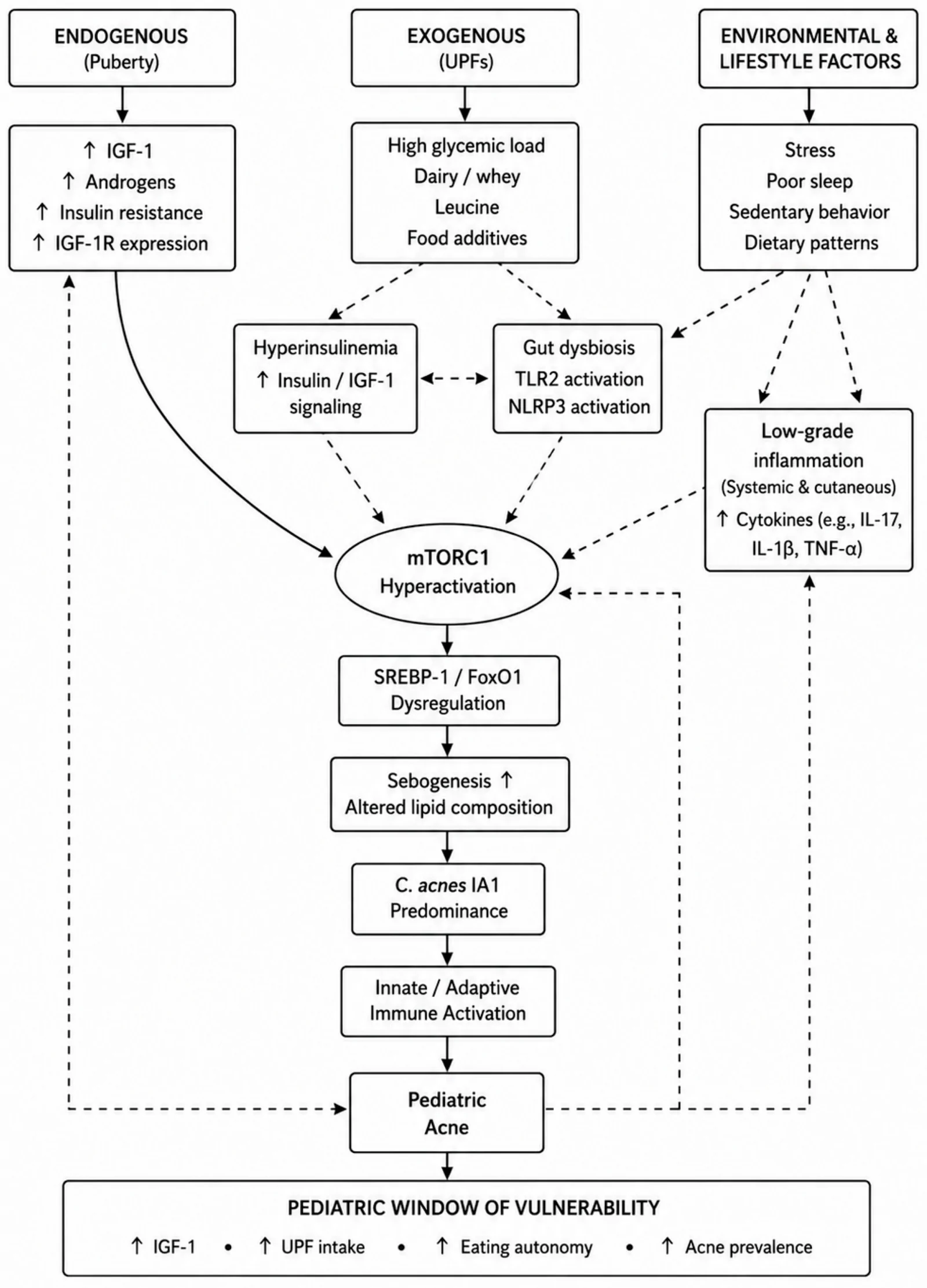
Proposed conceptual model of UPF-associated acne pathogenesis in pediatric populations. Endogenous factors associated with puberty, including elevated IGF-1, androgens, insulin resistance, and IGF-1R expression, may converge with exogenous ultra-processed food (UPF)-derived signals to drive mTORC1 hyperactivation. Downstream dysregulation of SREBP-1 and FoxO1 may contribute to sebogenesis and altered lipid composition, creating conditions favorable for *C. acnes* IA1 predominance and subsequent innate and adaptive immune activation, ultimately contributing to pediatric acne. This pathway is amplified during a proposed pediatric window of vulnerability, characterized by rising IGF-1 levels, increasing UPF intake, growing eating autonomy, and escalating acne prevalence. This figure was created using Biorender on 26 July 2026.

Although the core molecular mechanisms between acne pathogenesis in adults and adolescents are highly similar, adolescents show a unique period of physiologic susceptibility IGF-1 increase associated with puberty, insulin resistance, greater sebaceous gland responsiveness, and behavioral influences collectively amplifying these pathways. These considerations are highly relevant in the pediatric population, which is mechanistically primed even in the absence of dietary influences, due to hormonal fluctuations. Studies have found that IGF-1 and androgen levels increase during mid-puberty, and together these hormones activate the mTOR pathway, increasing acne lesions and sebogenesis [31,35]. In one study, circulating IGF-1 levels directly correlated with acne severity. In addition, as discussed, IGF-1 serves as a critical cofactor for androgen-mediated mTORC1 activation and subsequent cutaneous inflammation. Physiologic insulin resistance increases during puberty, contributing to compensatory hyperinsulinemia [73]. An additional feature of adolescent skin is the increased expression of IGF-1 receptors on immature sebocytes compared with mature sebocytes [31]. This suggests that adolescent skin is not only exposed to higher circulating IGF-1 levels but is also capable of increased responsiveness. Collectively, these physiologic changes may predispose pediatric patients to inflammatory acne, with dietary factors, such as UPFs, potentially exacerbating these underlying effects (Figure 2).

### 6.2. Behavioral Factors: UPF Consumption Patterns in Adolescents

Behavioral factors further strengthen the relationship between diet and acne in the pediatric population. As established in Section 4, US youths derive approximately 67% of their total caloric intake from UPFs, with increased consumption in adolescents compared to pre-school aged children [49,98]. Several factors contribute to this age-dependent rise. Increased autonomy and peer-influenced eating patterns allow adolescents to consume increased UPFs and snacks, without parental oversight [99,100,101]. Even structured environments, such as schools, contribute to this trend by offering UPFs as part of school lunches. One UK study found that 70% to 73% of calories of school lunches are derived from UPFs [102]. This poses an inherent problem as the daily environment where adolescents spend the majority of their day reinforces UPF consumption. Additionally, food marketing aggressively targets youth consumers, by utilizing their increased use of social media, susceptibility to social influence, and preference for hyperpalatable foods [98,103]. Critically, these dietary patterns occur at a time when IGF-1 and androgen surges are simultaneously occurring, leading to endogenous and exogenous mTORC1 activation.

### 6.3. Therapeutic Implications

These insights can guide dietary and nutritional education as an adjunctive therapy in acne management of the pediatric population. Limited real-world studies have emphasized a significant reduction in acne lesions with low-glycemic index diet [87,88]. Although additional high-quality studies, particularly in pediatric patients, are needed, these findings suggest that nutritional interventions may be an appropriate adjunctive therapy. Importantly, restrictive eating should not be promoted in this population, as overly restrictive eating patterns can increase the risk of eating disorders [104]. Instead, clinicians should encourage healthy eating patterns and balanced diets as an adjunct to standard treatments for acne. Discussions with families should emphasize habits that are sustainable over restrictive elimination to minimize the risk of disordered eating habits.

## 7. Literature Search Strategy

This narrative review was conducted using a non-systematic literature search of PubMed, MEDLINE, and EMBASE. This search included literature published from database inception through June 2025. Medical Subject Headings (MeSH) and key word texts were used to identify relevant literature. Search terms included combinations of “Acne Vulgaris,” “acne,” “ultra processed foods,” “processed foods,” “TOR Serine-Threonine Kinases (mTOR),” “Western diet,” “sebaceous glands,” “insulin-like growth factor-1 (IGF-1),” “*Cutibacterium acnes*,” and “skin microbiome.” There were no restrictions placed on publication date or location. Only English language articles were included. Clinical trials, original research articles, observational studies, meta-analyses, systematic reviews, and relevant mechanistic studies were considered for inclusion. Articles were selected based on their relevance to the topics discussed, with emphasis placed on mechanistic, epidemiologic, and clinical evidence pertinent to pediatric acne pathogenesis and dietary influences. When available, pediatric studies were prioritized. Due to limited pediatric specific studies on UPFs, adult clinical investigations were included to provide biological context. Boolean operators (AND/OR) were used to combine search terms, and representative search strategies, including variations of the search terms, are provided in Table 3. Because this was a narrative review, studies were selected based on relevance to the review objectives rather than through a formal systematic screening process.

## 8. Conclusions

The aforementioned evidence suggests a possible association between UPF consumption and acne pathogenesis in pediatric and adolescent populations. Through convergent activation of the insulin-IGF-1 axis, mTORC1 signaling, and inflammatory pathways, UPFs may create a systemic environment that potentiates acne pathogenesis. Pediatric populations may be especially exposed to these dietary patterns during periods of heightened hormonal flux, rendering them particularly susceptible to diet-mediated cutaneous dysregulation. Despite this, dietary intervention may represent an underutilized therapeutic target in pediatric dermatology.

Several limitations temper the strength of current conclusions. The lack of standardized dietary definitions of ultra-processed foods introduces significant variability, complicating cross-study comparisons. Furthermore, because UPFs encompass a variety of dietary components, it remains difficult to distinguish the relative contribution of individual components. In addition, the proposed pathway involving the insulin-IGF-1 axis, mTORC1 signaling, and downstream inflammatory cascades is biologically plausible; however, confirmation through well-designed pediatric studies is still needed. Importantly, pediatric-focused studies remain limited, leaving age-specific hormonal and metabolic dynamics insufficiently characterized. Finally, mechanistic human trials are notably lacking and represent a critical gap in the field.

Future research should prioritize several directions. Personalized nutrition frameworks that account for individual metabolic responses to dietary inputs may yield more targeted interventions. In addition, microbiome-targeted therapies could modulate systemic inflammatory signaling upstream of cutaneous manifestations. Validated downstream markers of mTORC1 activity should be incorporated into protocols to objectively quantify dietary impact on acne severity. Finally, the integration of evidence-informed dietary guidance into formal dermatology guidelines would represent a meaningful step toward holistic management of pediatric acne vulgaris. Future pediatric recommendations should emphasize family-centered counseling, balanced diet, and age-appropriate nutrition, while avoiding overly restrictive dietary practices.

## Figures and Tables

**Table 3:** Representative literature search strategies used in the narrative review.

Concept	Representative Search Strategy
Acne vulgaris	(“Acne Vulgaris” [MeSH] OR acne* [Title/Abstract])
Ultra-processed foods (UPFs)	(“Food, Processed” [MeSH] OR “ultra-processed food*” [Title/Abstract] OR “ultra processed food*” [Title/Abstract] OR “processed food*” [Title/Abstract] OR UPF [Title/Abstract])
mTOR signaling	(“TOR Serine-Threonine Kinases” [MeSH] OR mTOR [Title/Abstract] OR mTORC1 [Title/Abstract])
Insulin-like growth factor-1 (IGF-1)	(“Insulin-Like Growth Factor I” [MeSH] OR “IGF-1” [Title/Abstract] OR “IGF-I” [Title/Abstract] OR IGF1 [Title/Abstract] OR “insulin-like growth factor-1” [Title/Abstract])
Insulin	(“Insulin” [MeSH] OR insulin [Title/Abstract])
Western diet	(“Diet, Western” [MeSH] OR “Western diet*” [Title/Abstract])
Sebaceous glands	(“Sebaceous Glands” [MeSH] OR “sebaceous gland*” [Title/Abstract])
*Cutibacterium acnes*	(“*Cutibacterium acnes*” [MeSH] OR “*Cutibacterium acnes*” [Title/Abstract] OR “Propionibacterium acnes” [Title/Abstract])
Skin microbiome	(“Microbiota” [MeSH] OR “skin microbiome” [Title/Abstract] OR “cutaneous microbiome” [Title/Abstract] OR “skin microbiota” [Title/Abstract])

## Data Availability

No new data was created or analyzed in this study. Data sharing is not applicable to this article.

## Abbreviations

The following abbreviations are used in this manuscript:

UPF	Ultra-processed food
mTORC1	Mechanistic target of rapamycin complex 1
IGF-1	Insulin-like growth factor-1
IGFBP-3	Insulin-like growth factor binding protein-3
*C. acnes*	*Cutibacterium acnes*
SCFA	Short-chain fatty acid
HDAC	Histone deacetylase
TLR2	Toll-like receptor 2
TLR4	Toll-like receptor 4
NF-κB	Nuclear factor kappa B
AP-1	Activator protein 1
MAPK	Mitogen-activated protein kinase
MMP	Metalloproteinase
NLRP3	NOD-like receptor protein 3
GSDMD	Gasdermin D
IL	Interleukin
TNF-α	Tumor necrosis factor alpha
IFN-γ	Interferon gamma
PI3K	Phosphoinositide 3-kinase
Akt	Protein kinase B
SREBP-1	Sterol regulatory element-binding protein 1
FoxO1	Forkhead box protein O1
TSC	Tuberous sclerosis complex
Rheb	Ras homolog enriched in brain
S6K1	Ribosomal protein S6 kinase 1
PPARγ	Peroxisome proliferator-activated receptor gamma
HIF-1α	Hypoxia-inducible factor 1-alpha
AR	Androgen receptor
LXRα	Liver X receptor alpha
RXRα	Retinoid X receptor alpha
FASN	Fatty acid synthase
SCD	Stearoyl-CoA desaturase
FADS2	Fatty acid desaturase 2
MUFA	Monounsaturated fatty acid
NOVA	Not an acronym—classification system name
NHANES	National Health and Nutrition Examination Survey
P80	Polysorbate 80
CMC	Carboxymethylcellulose
LPS	Lipopolysaccharide
GPR41/43	G-protein-coupled receptor 41/43
AAD	American Academy of Dermatology
